# Pitfalls in Emergency Medicine: Survey-Based Identification of Learning Objectives for Targeted Simulation Curricula by Emergency Department Staff

**DOI:** 10.7759/cureus.11965

**Published:** 2020-12-08

**Authors:** Kerry-Lynn Williams, Tia S Renouf, Adam Dubrowski

**Affiliations:** 1 Family Medicine, Memorial University of Newfoundland, Happy Valley-Goose Bay, CAN; 2 Emergency Medicine, Memorial University of Newfoundland, St. John's, CAN; 3 Health Sciences, Ontario Tech University, Oshawa, CAN

**Keywords:** quality improvement research, medical education, emergency medicine

## Abstract

Introduction: The emergency department is a complex practice environment into which numerous factors may introduce both human and system error. Emergency physicians have to assemble and manage multidisciplinary teams with a moment’s notice to manage critically ill patients. The EM training programs across Canada are diverse with considerable variation among programs. Acquisition of both high acuity low occurrence (HALO) and crisis resource management (CRM) skills are crucial to the development of proficient emergency room physicians. Physicians and allied health workers were surveyed to identify potential causes of error in local emergency departments and to find simulation-driven solutions. Methods: An anonymous survey was prepared to evaluate potential pitfalls of emergency care in St. John’s, NL, Canada. It was distributed electronically to 108 medical staff, including physicians, nurses, and postgraduate year three (PGY3) residents. Respondents were asked about their experience with simulation education, and whether or not they feel that there is an opportunity for it in postgraduate emergency medicine training. Results: The response rate was 30%. Communication - with the emergency department team, consulting services, and patients - was identified as a potential topic for simulation, along with interruptions. Burnout, busy department, departmental crowding, end of shift handover, and incomplete/missing patient medical history were identified as topics that should be included in the emergency medicine curriculum. Following a review with the simulation expert panel, it was determined that end of shift handover could also be incorporated as a simulation in the existing curriculum.

Discussion: This survey looked at pitfalls in emergency medicine through a CRM lens. Six pitfalls were identified as important for patient safety, but not best addressed with simulation. These could be incorporated into the half-day curriculum as didactic lectures. Four important patient safety pitfalls were identified that could potentially be addressed with simulation and incorporated in the existing emergency medicine simulation curriculum.

## Introduction

The emergency department is a complex practice environment into which numerous factors may introduce both human and system error [1]. A great deal of healthcare is delivered by multidisciplinary teams. With this greater emphasis on management and leadership skills, there comes an increasing awareness of the importance of human factors in making changes to improve patient safety. Emergency physicians have to assemble and manage multidisciplinary teams with a moment’s notice to manage critically ill patients. This requires many non-clinical skills-termed crisis resource management skills (CRM). These CRM skills contribute to good team functioning and minimize the risk of errors [2]. Identifying potential errors, termed pitfalls, is important to implement preventative measures and corrective strategies. 

Simulation-based medical education has repeatedly been shown to favorably affect learner skills, knowledge and attitudes, and patient outcomes [3]. As such, it has become a crucial component of an emergency medicine postgraduate educational curriculum, typically focused on the development of high-acuity low-occurrence (HALO) skills. They are developed, taught, and reinforced with a number of modalities, including simulation, procedure sessions, academic rounds, journal club, point-of-care ultrasound, bedside teaching, and monthly textbook rounds. CRM, originally adapted from the aviation industry, focuses on effective team management [4]. CRM training addresses the non-technical skills necessary for effective teamwork [5].

Currently, in most postgraduate emergency medicine training programs across Canada, the educational curriculum has a large simulation component, largely driven by the need for HALO skills, with CRM skills infused in the learning process [6]. The infusion approach to teaching develops CRM skills by embedding them in the teaching of the set learning material [7]. Acquisition of both HALO and CRM skills are crucial to the development of proficient emergency room physicians.

Physicians and allied health workers were surveyed to identify potential causes of error in local emergency departments and to find simulation-driven solutions. The focus of the study was largely on CRM given that the current curriculum is predominantly driven by HALO skills with CRM skills infused within. A literature search was conducted, and an expert panel was consulted to develop an anonymous survey, which was then distributed to medical staff working in emergency departments in St. John’s, NL, Canada. Respondents were asked about their perceived practice pitfalls and their experience with simulation. Finally, results were analyzed and compared against the current Canadian College of Family Physicians - Emergency Medicine (CCFP-EM) training curricula across Canada.

## Materials and methods

A literature review was undertaken to identify classes of pitfalls. A keyword search was used in PubMed, Google Scholar, and Web of Science. As well, the medical subject headings (MeSH) terms “medication errors” and “emergency service, hospital” were utilized on PubMed. Articles were included if they were published within the past 15 years, in English, based on work that was undertaken in North America, and peer-reviewed. Both qualitative and quantitative studies were included. Following the literature review, a thematic analysis was undertaken which identified nine major categories of pitfalls. Individual informal consultations with a series of people-consisting of several emergency physicians, a research associate, and a medical simulation expert-further deconstructed categories into 16 specific pitfalls.

The 16 pitfalls included in the survey are summarized in Table 1 below.

**Table 1:** Nine Categories of Identified Pitfalls

Subcategory	Pitfall		
Bias	Anchoring bias (tendency to lock onto a diagnosis prematurely)	Confirmation bias (tendency to look for evidence to support a diagnosis)	Search satisficing bias (tendency to call off a search once something is found)
Decision Making	Diagnostic error	Medication error	
Department	ED crowding	Busy	
Patient	Communication with patients	Incomplete/missing patient medical history	
Personal	Burnout		
Shiftwork	Fatigue (during shift)		
Teamwork	Communication with allied health professionals (ED staff)	Communication with consulting services	
Transition	End of shift handover	Interruptions	
Triage	Undertriage (underestimating urgency of a patient’s condition upon arrival)		

An anonymous survey was prepared to evaluate potential pitfalls of emergency care in St. John’s, NL, Canada. The survey was developed and piloted using a committee consisting of emergency physicians and research associates. Respondents were asked about their experience with simulation education, and whether or not they feel that there is an opportunity for it in postgraduate emergency medicine training. The target audience for the survey was the emergency department staff - physicians, nurses, and residents. Clerkship medical students and off-service residents were not included due to limited exposure and experience. Ethics approval was obtained by the local Health Research Ethics Board (File #20162276) and the survey was distributed electronically to 105 medical staff.

Each of the 16 pitfalls was rated on a five-point Likert scale on (a) impact of pitfall on the daily work environment, and (b) impact of simulation training on addressing the pitfall. Data were compiled and frequently analyzed, identifying themes for simulation curricula. Pitfalls rated high (≥2.5) on both impact on work environment and potential impact of simulation could be included in a simulation curriculum. Pitfalls rated high (≥2.5) on the impact on the work environment and low on the potential impact of simulation could be included in a didactic curriculum.

**Figure 1:**
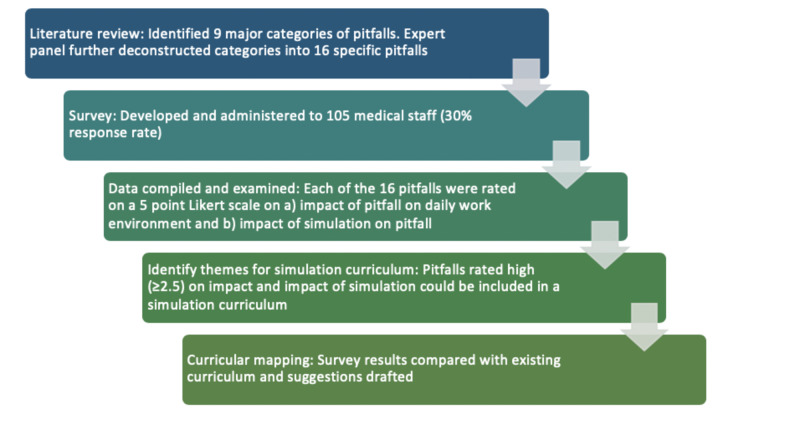
Summary of Methods

Finally, study results were compared with postgraduate CCFP-EM curricula, and results were compiled for dissemination and consideration. Methods are summarized in Figure 1.

## Results

**Figure 2:**
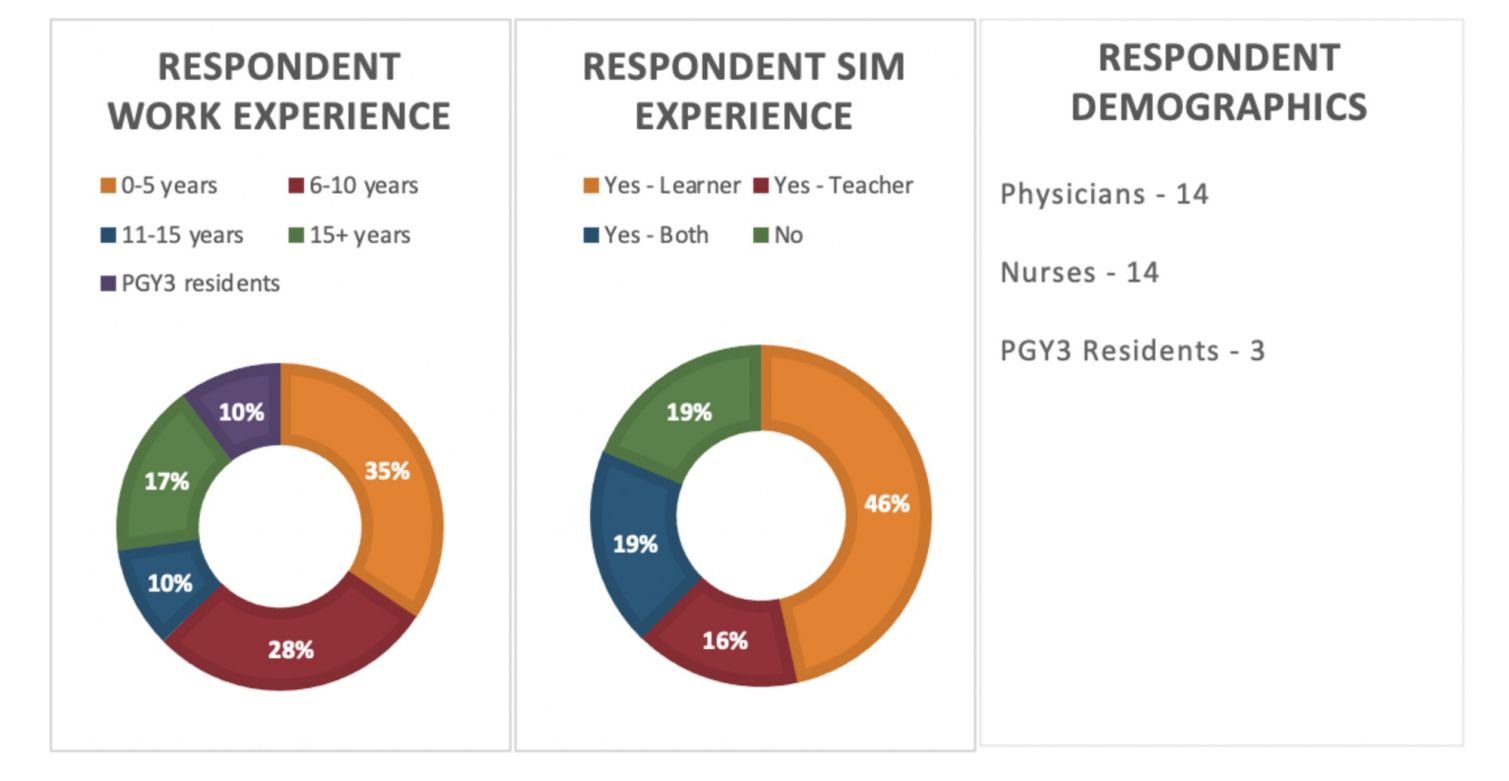
Respondent Demographics Information PGY: postgraduate year

The response rate was 30%. Demographics of respondents are illustrated in Figure 2. Based on the combined Likert scale rankings, an average rating of 2.5 or higher (out of five) was considered to be “significant”. Pitfalls were therefore correlated with their corresponding potential benefit of a targeted simulation curriculum. Therefore, a high impact pitfall with a potentially high impact targeted simulation curriculum was considered to be covered as part of the simulation curriculum in the postgraduate year three (PGY3) emergency medicine enhanced skills program. Similarly, pitfalls that were high impact but corresponded with a low impact use of simulation were recommended to be incorporated in the didactic portion of the emergency medicine curriculum.

**Figure 3:**
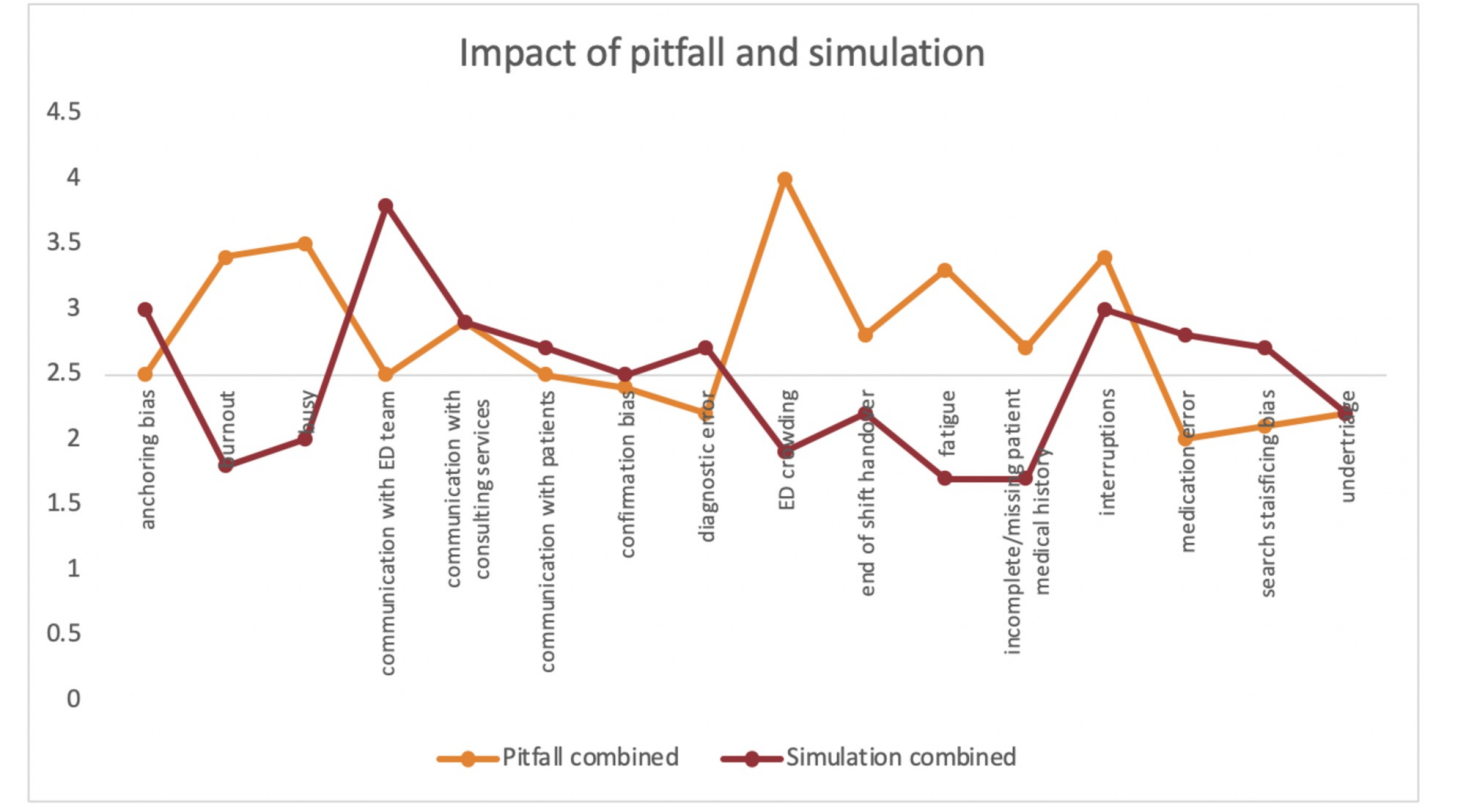
Combined Impact of Pitfall and Simulation

The survey focused primarily on crisis resource management skills, however, there was a section where respondents could identify other pitfalls not included in the survey. No HALO skills were identified as pitfalls. Finally, a panel of simulation experts reviewed survey results and the appropriateness of potential simulation scenarios. Survey results are summarized in Figure 3. Communication was identified as a potential topic for simulation, along with interruptions. Burnout, busy department, departmental crowding, end of shift handover, and incomplete/missing patient medical history were identified as topics that should be included in the emergency medicine curriculum. Following a review with the simulation expert panel, it was determined that end of shift handover could also be incorporated as a simulation in the existing curriculum.

## Discussion

This survey looked at pitfalls in emergency medicine through a CRM lens. Six pitfalls were identified as important for patient safety, but not best addressed with simulation. This included burnout, busy department, ED crowding, end of shift handover, incomplete/missing patient history, and fatigue. These could be incorporated into the half-day curriculum as didactic lectures. Four important patient safety pitfalls were identified that could potentially be addressed with simulation and incorporated in the existing emergency medicine simulation curriculum. These included communication with the ED team, communication with consulting services, communication with patients, and interruptions. The EM training programs across Canada are diverse with considerable variation among programs [6,8]. That being said, most simulation curricula focus on a mixture of must know/core/common skills, as well as infrequent presentations that may not be seen during the training program but that are essential to know about should they present to the department. This type of skill is often referred to as a HALO skill. HALO skills are essential to have and maintain in the emergency department. They are developed, taught, and reinforced with a number of modalities, including simulation, procedure sessions, academic rounds, journal club, point-of-care ultrasound, bedside teaching, and monthly textbook rounds. Because CRM skills are often infused in technical simulation scenarios, the survey results would be relatively easy to incorporate in a pre-existing curriculum, with expansion during the debriefing session. As well, emergency department-specific challenges, such as a busy, crowded department with numerous interruptions, can also be incorporated in a “technical” simulation scenario through the use of actors, standardized patients, and noise distractions. Another way to simulate the hustle and bustle of the department would be to complete an *in-situ* simulation in the department itself. Of course, logistically this is much more challenging, however, it has been shown to have favorable outcomes [9-13].

Recent reviews have outlined the importance of teamwork, effective communication, and leadership behavior in managing emergency situations, leading to the inclusion of CRM into pediatric advanced life support (PALS), advanced cardiac life support (ACLS), and neonatal resuscitation program (NRP) teaching programs [14-16]. As such, crisis resource management is starting to be infused into postgraduate emergency medicine curricula through the use of simulation-based educational content such as cardiac arrest and resuscitation scenarios [6].

Limitations of this study include a low response rate (30%) and a survey focused primarily on CRM. Although the assumption was that because HALO skills are a large focus of postgraduate EM simulation curricula, they will likely not represent a large proportion of perceived pitfalls. However, they should have been included in the original survey. There was a free text box to include any other perceived pitfalls, which did not result in any HALO skills being identified, but the original survey should have included a combination of both. The emergency departments included in the study were only in one province, and thus may not be generalizable to pitfalls of emergency care across the country. Future work could include expansion of the project to include other centers as well as validation of the survey to ensure reliability.

## Conclusions

Excessive pressures on the health care system pass directly to and through the emergency department, adding to an already complex practice environment. As a result, there are many pitfalls in emergency medicine. This study identified four, in particular, that could be targeted with simulation-augmented training and six that could be addressed in didactic lectures. Canadian postgraduate emergency medicine educational curricula consist of both didactics and simulation. This research informs both and could theoretically easily be incorporated into existing emergency medicine curricula moving forward.
